# Coupling Raman, Brillouin and Nd^3+^ Photo Luminescence Spectroscopy to Distinguish the Effect of Uniaxial Stress from Cooling Rate on Soda–Lime Silicate Glass

**DOI:** 10.3390/ma14133584

**Published:** 2021-06-26

**Authors:** Michael Bergler, Kristian Cvecek, Ferdinand Werr, Alexander Veber, Julia Schreiner, Udo R. Eckstein, Kyle G. Webber, Michael Schmidt, Dominique de Ligny

**Affiliations:** 1 Institute of Glass and Ceramics, Friedrich-Alexander-Universität Erlangen-Nürnberg, Martensstrasse 5, D-91058 Erlangen, Germany; ferdinand.werr@fau.de (F.W.); julia.schreiner@fau.de (J.S.); udo.eckstein@fau.de (U.R.E.); kyle.g.webber@fau.de (K.G.W.); 2 Institute of Photonic Technologies, Friedrich-Alexander-Universität Erlangen-Nürnberg, Konrad-Zuse-Strasse 3/5, D-91052 Erlangen, Germany; Kristian.Cvecek@lpt.uni-erlangen.de (K.C.); michael.schmidt@lpt.uni-erlangen.de (M.S.); 3 Erlangen Graduate School in Advanced Optical Technologies (SAOT), Friedrich-Alexander-Universität Erlangen-Nürnberg, Paul-Gordan-Strasse 6, D-91052 Erlangen, Germany; 4 Department of Chemistry, Humboldt-Universität zu Berlin, Brook-Taylor-Strasse 2, D-12489 Berlin, Germany; alexveb@gmail.com

**Keywords:** glass structure, Raman spectroscopy, Brillouin spectroscopy, soda–lime silicate glass, window glass, fictive temperature, cooling rate, uniaxial stress

## Abstract

Evolution of spectroscopic properties of a soda–lime silicate glass with different thermal history and under applied uniaxial stress was investigated using Raman and Brillouin spectroscopies as well as Nd^3+^ photoluminescence techniques. Samples of soda–lime silicate with a cooling rate from 6 × 10^−4^ to 650 K/min were prepared either by controlled cooling from the melt using a differential scanning calorimeter or by a conventional annealing procedure. Uniaxial stress effects in a range from 0 to −1.3 GPa were investigated in situ by compression of the glass cylinders. The spectroscopic observations of rearrangements in the network structure were related to the set cooling rates or the applied uniaxial stress to calculate an interrelated set of calibrations. Comparing the results from Raman and Brillouin spectroscopy with Nd^3+^ photoluminescence analysis, we find a linear dependence that can be used to identify uniaxial stress and cooling rate in any given combination concurrently. The interrelated calibrations and linear dependence models are established and evaluated, and equations relating the change of glass network due to effects of cooling rate or uniaxial stress are given.

## 1. Introduction

The response of the glass network to external factors such as chemical alterations, thermal influence or stress—externally applied or residual—is not as well understood as it should be. Models and calibrations of these external factors are well established based on their typical influence on the glass network alone. However, in general, an extensive combination of different factors of influence affects the network structure simultaneously, often due to comparable effects on the general properties such as density or refractive index while providing distinct changes of the atomic arrangements. This study monitors changes of the network structure using luminescence and vibrational spectroscopic observations. Assigning changes in the spectroscopic observations to a change of cooling rate and applied uniaxial stress, an interrelated set of calibrations is calculated. A comparison of these calibrations is used to establish models to distinguish between the effects of the cooling rate and the uniaxial stress and determine both values quantitatively.

In the past decade, the processing of glass was extended by a vast range of new methods, especially by laser-based cutting [1,2], additive manufacturing [3,4,5] or direct structuring [6,7]. All these methods, also including more conventional approaches like molding [8], utilize temperature as a core element. The influence of temperature is often locally limited, and the subsequent cooling process is uncontrolled. This results in a rearrangement of the glass network based on a locally dependent cooling rate, affecting such properties of the glass as density or refractive index [9,10]. A locally rearranged glass network structure can further induce residual stress in the surrounding volume affecting the elastic properties of the bulk glass [11]. To understand how either the cooling rate or residual stress influence the behavior of the glass network, it is necessary to analyze both effects separately [12]. A known thermal history of a glass can be easily induced in the material by either a controlled cooling of the glass from a temperature well above the glass transition temperature (*T_g_*), or by a conventional annealing process. Samples from both processes can be easily studied ex situ [13,14]. The influence of residual stress can be evaluated using isostatic or uniaxial compression tests monitoring the response of the glass network in situ or after treatment to a certain maximum pressure inducing permanent densification [15,16,17]. The changes in the glass network can be observed non-destructively using commonly used vibrational spectroscopy methods like Raman scattering and Fourier-transform infrared (FTIR) spectroscopies, as well as less common photoluminescence or Brillouin spectroscopy techniques. Residual stress or different cooling rates often assert themselves in a similar change of the spectroscopic properties, complicating the separation of these two effects. Therefore, a method to distinguish between both effects and to further analyze them quantitatively, is highly advantageous to assess the residual effect imparted by the manufacturing process. In this paper, we observe soda–lime silicate since it is the most prevalent type of glass in many aspects of everyday life as windowpanes or glass containers and accounts for the largest quantity of human-made glass [18].

In our work, we analyze glass samples with different cooling rates ex situ and uniaxial compression of the material in situ using Raman, Brillouin and photoluminescence spectroscopies. This paper aims at the definition of an interrelated set of calibrations for soda–lime silicate capable to determine the glass cooling rate and the uniaxial stress in the elastic regime. This is achieved by relating observations of inelastic light scattering (Raman and Brillouin spectroscopies) and photoluminescence spectroscopy of Neodymium (Nd^3+^) to different cooling rates and applied uniaxial stress. Alternative calibration methods linking the effect of cooling rate and stress, using permanent densification, were already reported for soda–lime silicates using the mentioned spectroscopic techniques separately. Comparing our interrelated calibrations to each other, we provide a method to separate the effects of cooling rate or uniaxial stress on the glass network structure simultaneously in any possible combination. In order to determine the cooling rate in a range from 6 × 10^−4^ to 650 K/min or the uniaxial stress in a range from 0 to −1.3 GPa, high-resolution inelastic light scattering experiments were performed. Additionally, the natural contamination of soda–lime silicates with rare-earth element (REE) Nd^3+^ is utilized as one more sensor of the cooling rate and uniaxial stress by observing its near-infrared photoluminescence spectrum corresponding to the ^4^F_3/2_→^4^I_9/2_ transition. Calibrations were performed and compared to the ones available in literature to quantify structural changes due to a change of cooling rate or applied uniaxial stress. Equations relating specific observables deduced from the inelastic light scattering experiments to the cooling rate and the uniaxial stress are determined. Using these interrelated calibrations, we compare the observables deduced from inelastic light scattering experiments to the photoluminescence of Nd^3+^, concluding a linear combination between the effects of cooling rate and residual stress. The comparison of observables versus each other is described using multiple linear regression to establish models to quantify the independence of contributions of cooling rate or uniaxial stress on specific observables and quantitatively estimate the cooling rate and uniaxial stress simultaneously in any possible combination.

## 2. Materials and Methods

Commercially available OptiWhite soda–lime silicate glass (Pilkington Ltd., Lancashire, UK/NSG Co. Ltd., Tokyo, Japan; Composition [mol.%]: 71.3 SiO_2_, 12.4 Na_2_O, 9.3 CaO, 6.3 MgO, 0.3 Al_2_O_3_, 0.3 K_2_O, 0.1 SO_3_, 0.01 Fe_2_O_3_) was used for the experiments. OptiWhite is often referred to as iron (Fe) reduced window glass, with several of its properties listed in [19].

### 2.1. Correlation of Cooling Rate to the Fictive Temperature

The glass is metastable. It needs at least a complementary order parameter to describe its state [20], which is affected both by its cooling rate and by long heat treatment at a given temperature. The simplest way, proposed by Tool et Eichlin [21], is to introduce the fictive temperature (*T_f_*) as an extra order parameter. This approach is phenomenological and was found to work perfectly for many properties as heat capacity, refractive index or stress [22,23,24]. In order to determine the fictive temperature, the point where structural relaxation does not have enough time to take place, the cooling rate (*q_g_*) needs to be related to the viscosity in the domain of the glass transition. The liquid is considered as a Maxwell body; its relaxation time (*τ_r_*) is related to the shear modulus (*G_∞_*) and the viscosity (*η*) by the following Equation (1):

(1) $$\tau_{r}=\frac{\eta }{G_{\infty }}$$

Using [25,26,27], numerical values obtained from [19] and complementary values from SciGlass database, *q_g_* can be directly related to *T_f_* using the viscosity-temperature dependence with:

(2) $$\begin{array}{ll} \log\left(q_{g}\left(T_{f}\right)\right) & =13-\log_{10}\left(\eta \left(T_{f}\right)\right)\\ & =13-\left(A+\frac{B}{\left(T_{f}-T_{0}\right)}\right)\\ & =13-\left(-2.84+\frac{4637}{\left(T_{f}-244.71\right)}\right) \end{array}$$

where *q_g_* is in K/min, *η* in Pa·s, *T_f_* and *T*_0_ in °C. The three variables *A*, *B* and *T*_0_ are coefficients of the traditional Vogel–Fulcher–Tammann (VFT) equation. The significance of both parameters, *T_f_* and *q_g_*, is equivalent, and both concepts can be used indifferently. This means that equivalent glass states can be achieved either by controlling *q_g_* or after heat treatment at the corresponding *T_f_* longer than *τ_r_* to allow a full relaxation (here the heat treatment time was longer than 3*τ_r_*). Further on in this article only the *q_g_* will be used, as it is a more illustrative parameter that *T_f_*.

### 2.2. Generation of Glasses with Different Cooling Rates

Samples with different cooling rates were obtained using the differential scanning calorimeter (Perkin Elmer DSC 8500) included in the ARABICA (Associated Raman and Brillouin Calorimeter) setup [28]. Two series of small glass discs (*q_g_*1 and *q_g_*2), 5 mm in diameter and 1 mm in height, were heated in a platinum crucible to 650 °C, well above the glass transition temperature (*T_g_* = 558 °C). This procedure ensures clearing the samples of their thermal history completely, and the cooling process can be controlled through the complete glass transition range. The subsequent cooling of the samples to room temperature was performed with a constant cooling rate controlled between 0.5 and 650 K/min. Due to the small size of the samples and their large exchange surface area, a constant cooling rate is achieved through out the full samples, which are then free from residual stress. By this way, two glass discs for each cooling rate were produced. An additional low fictive temperature (i.e., equivalent low cooling rate) sample was produced using a conventional annealing method [29]. The sample was isothermally treated by placing it in a platinum crucible in an annealing furnace, which was kept at a constant temperature below *T_g_* at 488 °C (±1 °C) [30]. The annealing time was chosen to be three times the calculated relaxation time (3*τ_r_*) using Equation (1), i.e., 432 h. After isothermal treatment the sample was directly quenched in deionized water. This fast cooling ensures that the prepared glass sample will have the desired *T_f_*, which equals the annealing temperature, if the cooling time at the end of the isothermal treatment is significantly shorter than the relaxation time. This annealing temperature being well below *T_g_*, the quenching acts only on the elastic response of the glass that remains residual stress free. Using Equation (2), an equivalent cooling rate of 6 × 10^−4^ K/min is determined.

### 2.3. In Situ Observation during Uniaxial Compression

**Figure 1:**
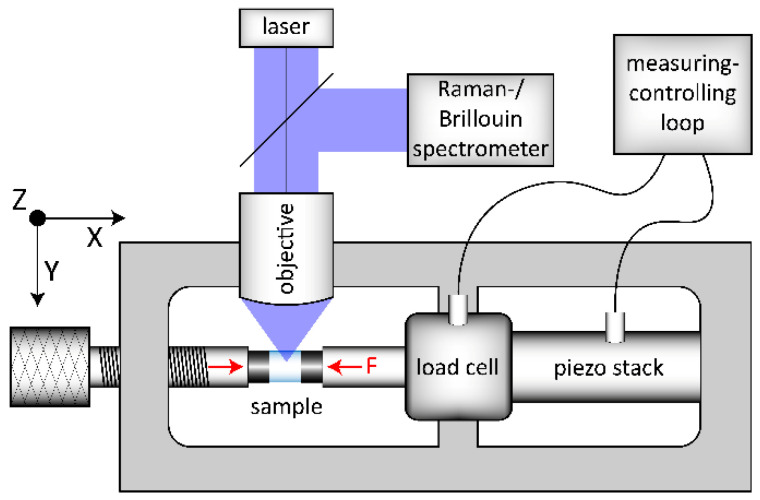
Scheme of the experimental setup used for the in situ uniaxial compression experiments. The load frame setup is arranged in the x–y plane and the excitation laser is incident from z-direction. The left compression piston uses a mechanical screw system for preloading of the sample (left red arrow). The right compression piston (right red arrow, F) is connected to the load cell and the piezo stack. This piston, integrated in a measuring–controlling loop, is used for high accuracy (±0.001 GPa) loading and constant conditions during observation (F).

OptiWhite glass cylinders with 1.8 and 3.8 mm in diameter and 4 mm in height were used for in situ observations during uniaxial compression. Prior to the densification experiments and following a conventional annealing method [24], two series of cylinders (US_T_g_1 and US_T_g_2) were annealed at *T_g_* to release any residual stress and quenched in deionized water to room temperature resulting in a cooling rate of 10 K/min. A third cylinder (US_annealed) was annealed at 488 °C (±1 °C) for 432 h until relaxation, which is equal to a cooling rate of 6 × 10^−4^ K/min, see Section 2.2. The front surfaces of all cylinders were plane parallel and polished to optical standards. The cylinder walls were polished to a grain size of 15 µm to ensure a small crack depth, preventing a possible crack initiation at low compressive stress. For compression testing, an in-house built load frame was used, shown schematically in Figure 1. The setup is built in an open 2D-arrangement (*x*-*y* plane) to allow in situ observation in *z*-direction. The left compression piston (mechanical screw system) is used for preloading the sample with a certain uniaxial force. Further loading is implemented electronically using the piezo stack, which is included in a measuring-controlling loop together with the load cell. Thus, a controlled loading situation and a constant compression condition can be achieved up to 10 kN resulting in a maximum possible uniaxial stress of −0.88 and −1.3 GPa for the cylinders of 3.8 and 1.8 mm in diameter, respectively. The uniaxial stress sign follows the convention used in mechanics where negative values correspond to compressive stress and positive to tensile one. In doing so, we want to prevent the readers from confusing the uniaxial stress used in this study from more common investigations done under hydrostatic pressure conditions.

The OptiWhite samples (see Figure 1 light blue cylinder, sample) were stressed in-between tungsten-carbide cylinders (see Figure 1 dark grey cylinders) with all surfaces polished to optical standards. The flat surfaces of the tungsten-carbide cylinders were in contact with the sample, the other slightly rounded surfaces were in contact with the flat compression pistons. This arrangement ensures a uniaxial stress (*US*) propagation, compensating small angle and alignment mismatches. Observations were collected at constant uniaxial compression conditions from 0 to −1.3 GPa (±0.001 GPa) during loading and unloading.

### 2.4. Vibrational Analysis

The vibrational response of the glass network was observed using the in-house built ARABICA experimental setup [28]. The optical and acoustic phonons vibrations of the glass network at the laser focal point were measured using a single-frequency, 488 nm, 100 mW CW laser source (Coherent Sapphire SF). The focusing objective was an OptoSigma PAL-50-L (50×, NA 0.42, WD 20.5 mm). The measurement setup was built in a 180° reflective way, so the scattered light contains the Rayleigh (elastic scattered light), the Raman (inelastic-optical-phonons scattered light) and the Brillouin (inelastic-acoustic-phonons scattered light) contributions. The Raman signal was observed with a CCD camera after passing a monochromator with a high spectral resolution grating (1800 lines/mm). The Brillouin signal was analyzed using a tandem Fabry–Perot interferometer (TFP). Veber et al. [28] published the exact specifications and a precise description of the complete setup.

Nd^3+^ photoluminescence was observed using a Thermo Fisher Nicolet Almega XR spectrometer. The spectrometer was used with a 780.42 nm CW laser source for excitation and a grating for wide spectral range (600 lines/mm). The focusing objective was the same OptoSigma PAL-50-L.

In order to compare the acquired spectra almost independently of the acquisition conditions and possible noise, reducing the stability and reproducibility of common Gaussian fit approaches, we identified initially several observables with high sensitivity and reproducibility to cooling rate and stress. These observables were then selected for subsequent analysis. For the vibrational analysis in our work, we focus on four different observables, which will be described in detail further below. The first two observables are obtained from the Raman signal: the *σ*-parameter of the main band (*σ_MB_*, 1st observable) and the *maximum position* of the Q-range (*MP_Q_*, 2nd observable). The 3rd observable is the *Brillouin shift* (*BR*). The last observable is the *σ-*parameter of the Nd^3+^ luminescence (*σ_Nd_*, 4th observable).

#### 2.4.1. Raman Spectroscopy

**Figure 2:**
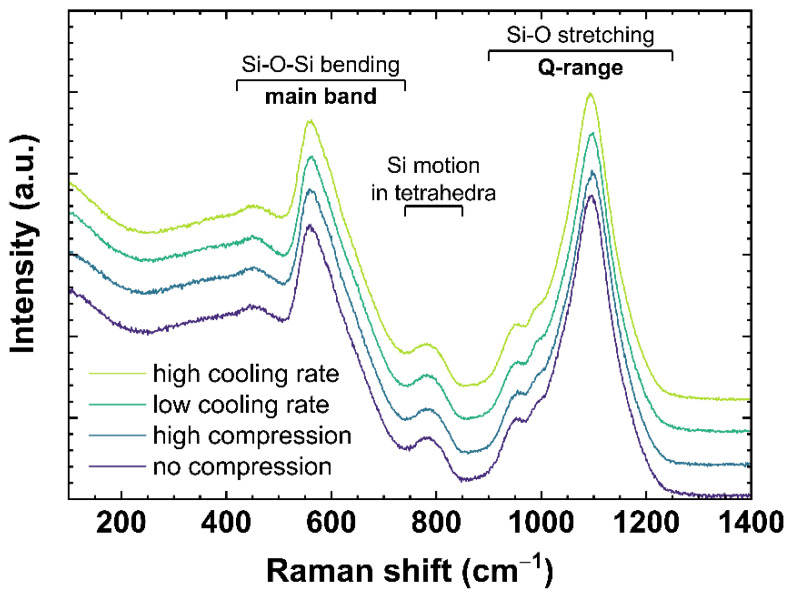
Raman spectra of OptiWhite *q_g_*1 cooled with 6 × 10^−4^ K/min (low cooling rate) and 650 K/min (high cooling rate) as well as US_T_g_1 under no uniaxial compression (0 GPa) and high uniaxial compression (−0.85 GPa). Assignments of the observed vibrational bands are given in Section 2.4.1.

Raman spectra of OptiWhite were collected ex situ after cooling the melt at different rates or in situ under different uniaxial stress. Figure 2 shows typical Raman spectra for soda–lime silicates ranging from 100 to 1400 cm^−1^. The spectra are normalized to the total area of the spectrum in the frequency range from 100 to 1400 cm^−1^ (total area = 1) with no baseline correction performed. The slow-cooled and fast-cooled samples were subjected to a cooling rate of 6 × 10^−4^ and 650 K/min, respectively, as described in Section 2.2. The spectra under compression are US_T_g_1 observed at 0 (no compression) and −0.85 GPa (high compression) as described in Section 2.3. According to the literature [31,32,33,34,35], the bands ranging from 420 to 740 cm^−1^ (referred to as main band, *MB*) are associated with Si–O–Si bending vibration modes. The 600 cm^−1^ band is often also attributed to the three-Si membered rings breathing mode [34] similar to the D_2_ defect band in fused silica [36,37]. The Si motion in its tetrahedral oxygen cage is associated with the 800 cm^−1^ band [33]. The bands between 850 and 1200 cm^−1^ (referred to as Q-range, *Q*) correspond to Si–O stretching vibration modes of *Q^n^* species [38]. Following [39], the Q-range can be assigned as: *Q*^4^~1200 cm^−1^; *Q*^3^~1098 cm^−1^; *Q*^2^~950 cm^−1^; and *Q*^1^~900 cm^−1^. The number at the exponent is referring to the number of non-bridging oxygen per SiO_4_ tetrahedra.

The bands and therefore the associated vibration modes are known to be sensitive to permanent densification [40] or a change of cooling rate [41]. In the case of permanent densification, [40] reported a change in *MB* maximum from 420 to 740 cm^−1^. With increasing densification, the barycenter shifts to higher wavenumbers indicating a *Q*^2^ formation at the expense of *Q*^3^ since the peak at 560 cm^−1^ decreases while the peak at 600 cm^−1^ increases. For a change of cooling rate, [41] reported a change in *MB* and Q-range. They observed an increase of intensity for the 600 and 950 cm^−1^ peaks with increasing cooling rate. To evaluate the changes of the main band a linear baseline was subtracted anchored to 500 and 730 cm^−1^. This area of interest was extracted from the whole spectrum and normalized to the total area (total area = 1). The *σ*-parameter (*σ_MB_*) defined by Deschamps et al. [42] was calculated as follows:

(3) $$\frac{\int_{\alpha 1}^{\sigma_{MB,Nd}}I_{Raman}\left(\alpha \right)\;d\alpha }{\int_{\alpha 1}^{\alpha 2}I_{Raman}\left(\alpha \right)\;d\alpha }=\frac{1}{2}$$

where *α*1 is the lower border (500 cm^−1^) of the integral and *α*2 the higher border (730 cm^−1^) of the integral. *I_Raman_* indicates the intensity of the signal at the Raman shift *α*.

For the evaluation of the Q-range a linear baseline was subtracted anchored to 855 and 1300 cm^−1^. After extraction and normalization of the area, the *maximum position* for the Q-range (*MP_Q_*) was calculated at the maximum intensity of this range. Therefore, the band centered at 1100 cm^−1^ was fitted using a 9th order polynomial between 1050 and 1130 cm^−1^ to denoise the spectrum and minimize the calculation error of the *MP_Q_*. Conventional peak deconvolution of the Q-range was attempted, but it requires a larger number of parameters and shows a poor reproducibility.

To compare this study with existing literature, the evaluation of the main band, regarding the baseline subtraction and the lower and higher borders for calculating the *σ*-parameter, was kept identical as described in [40]. The evaluation of the main band using the *maximum position* or the distribution’s mean, [37] shows comparable observations of the *σ*-parameter, but with higher noise and poorer stability of the method. For the evaluation of the Q-range, the *maximum position* was chosen, due to comparison reasons with literature [41]. Furthermore, using the *σ-*parameter method for the Q-range shows a lower signal to noise ratio and poorer reproducibility.

#### 2.4.2. Brillouin Spectroscopy

**Figure 3:**
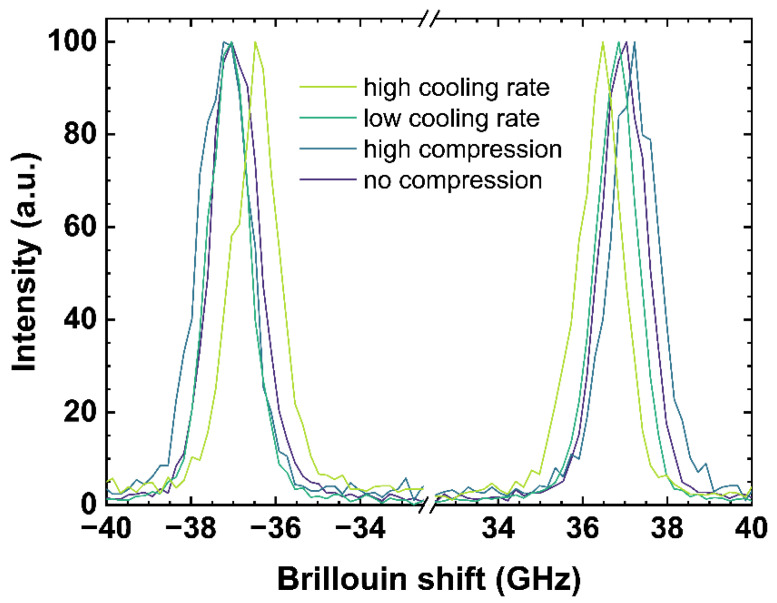
Brillouin spectra of OptiWhite *q_g_*1 cooled with 6 × 10^−4^ K/min (low cooling rate) and 650 K/min (high cooling rate) as well as US_T_g_1 under no uniaxial compression (0 GPa) and high uniaxial compression (−0.85 GPa). Negative shifts are related to the anti-Stokes signal (**left**) and positive shift to the Stokes signal (**right**), respectively.

An incident laser on the measured sample generates acoustic vibrations parallel to the incident laser direction. The incident laser is diffracted at the wave fronts of the induced vibrations, corresponding to the speed of sound of the observed material. The constructive interference of the resulting reflection at the (volume) grating, moving at the speed of sound (toward and away from the observer), is measured as Brillouin shift. Figure 3 contains examples of Brillouin spectra of the same samples and from the same measurement location as used in Figure 2, since both observations were collected simultaneously.

Figure 3 shows both the negative (anti-Stokes signal) and the positive (Stokes signal) Brillouin shifts. The anti-Stokes and Stokes signal were fitted using a Gaussian model and the arithmetic mean values of both signals were calculated to determine the *Brillouin shift* (*BR*). A difference in pressure or a change of the cooling rate rearranges the glass network structure and modifies the elastic constant associated to the speed of sound in the glass network inducing a shift in the observed Brillouin scattering bands [17,43]. High compression as well as a low cooling rate causes a Brillouin shift to higher frequencies due to a densification of the network structure.

Brillouin shift is a function of the geometry and the laser excitation wavelength (*λ*). The calibrations given here below were obtained in backscattering geometry using a 488 nm laser and thus observing the Brillouin shift of the longitudinal mode. The conversion to any excitation *λ* can be easily made by multiplying *BR* by the ratio *λ*/488 nm. A direct determination of the longitudinal velocity or the comparison between different geometries will require the knowledge of the refractive index. The specific equations can be found for example in [44].

### 2.5. Neodymium^3+^ (Nd^3+^) Luminescence

As it was already mentioned above, OptiWhite contains trace amounts of rare-earth elements (REE). In most soda–lime silicates, Nd_2_O_3_ is used as a decoloring agent or is included naturally in the raw materials. The inclusion of trace amounts of REE (few ppm) does not modify the glass structure, but their photoluminescence can be used as sensor of changes happening in the glass network induced by variation of thermal history and applied stress. In particular, the ^4^F_3/2_→^4^I_9/2_ optical transition of Nd^3+^ ions results in emission within the spectral range between 840 and 950 nm, depending on the host material [45,46,47]. Compared to crystalline materials, Nd^3+^ has no specific substitutional REE-site in glasses resulting in a broad emission spectrum. The ^4^F_3/2_→^4^I_9/2_ transition is a sensitive quadrupole transition in the 4f-shell being responsive to changes in the surroundings of Nd^3+^, although the intra-configurational 4f-shell transition is well shielded from the outside [48,49]. Therefore, changes in the local ion environment will affect the Nd^3+^ photoluminescence spectrum associated with this transition. The changes could be of chemical nature, residual stress, densification [50,51], or variation of the glass thermal history [52].

**Figure 4:**
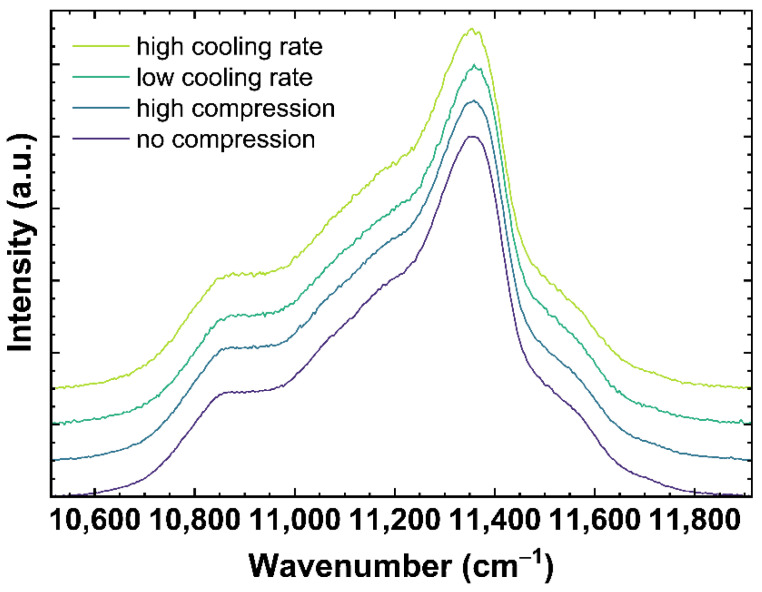
Neodymium^3+^ luminescence spectra of OptiWhite excited at 780.42 nm. The displayed spectra show *q_g_*1 cooled with 6 × 10^−4^ K/min (low cooling rate) and 650 K/min (high cooling rate) as well as US_T_g_1 under no uniaxial compression (0 GPa) and high uniaxial compression (−0.85 GPa).

Figure 4 shows examples of Nd^3+^ luminescence spectra excited at 780.42 nm. The spectra correspond to the same samples as shown in Figure 2 and Figure 3. Modifying the network structure to a more densified state, either due to compression or lower cooling rates, is expected to decrease the distance Nd-O and, following the nephelauxetic effect, shifts the observed Nd^3+^ bands to lower wavenumbers [46,47].

To evaluate the observed shift of the Nd^3+^ bands the *σ*-parameter (*σ_Nd_*) was calculated as described in Section 2.4.1 (see Equation (3)). The collected spectra were corrected with a linear baseline anchored to 10,513 and 11,913 cm^−1^ and normalized to the total area. The *σ_Nd_* was integrated in the range from 10,513 to 11,913 cm^−1^ (see α1 and α2 in Equation (3)).

## 3. Results and Discussion

In this section, several calibrations are determined. Instead of using the usual linear equation writing, we prefer to write the equations in the form:

(4) $$Physical\;variable=\frac{Observable-Offset}{Rate}$$

The value of the *Offset* results from the value of the *Observable* when the *Physical variable*, such as *US* or log(*q_g_*), is equal to zero. Since some small calibration variations can happen from one experimental setup to another, our determined value of the *Offset* can be easily modified to take them in account.

### 3.1. Calibration of the Raman Response

**Figure 5:**
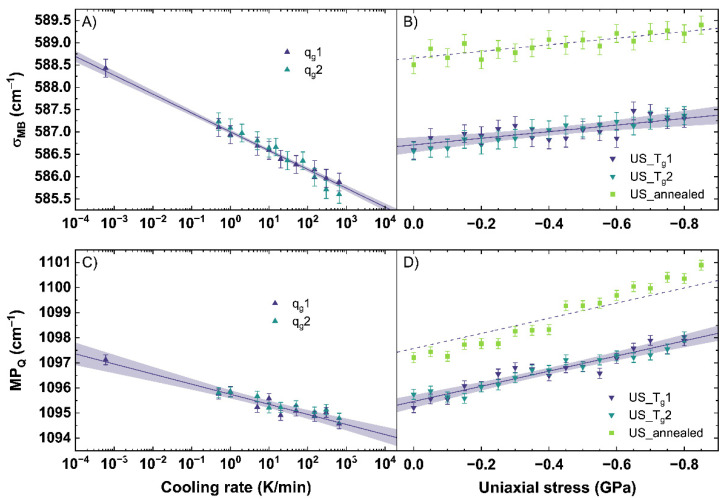
(**A**) *σ*-parameter of the main band (*σ_MB_*) for a change of cooling rate (*q_g_*) and (**B**) under different uniaxial stress (*US*). (**C**) *maximum position* of the Q-range (*MP_Q_*) for a change of *q_g_* and (**D**) under different *US*. *q_g_*1 and *q_g_*2 are indicated by the dark blue and cyan up triangles. US_T_g_1 and US_T_g_2 are displayed by the dark blue and cyan down triangles. US_annealed is indicated by light green squares. The shaded areas show the 95% confidence interval of the linear fits.

The obtained *σ_MB_* and *MP_Q_* were calculated from the Raman spectra, evaluated for all observed conditions (different cooling rates, uniaxial stress), and are shown in Figure 5. A measurement uncertainty of 0.2 cm^−1^ was calculated from the resolution of the used CCD camera of the Raman spectrometer and the calculation error evaluating the spectra. Figure 5A shows the behavior of *σ_MB_* for different cooling rates. *q_g_*1 and *q_g_*2 show a linear trend to lower wavenumbers with increasing cooling rate. A similar trend is observable for the *MP_Q_* displayed in Figure 5C.

**Figure A1:**
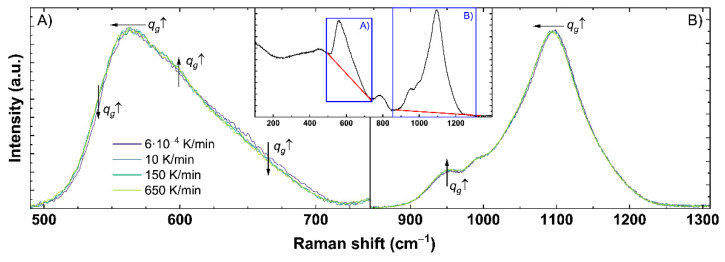
Detailed analysis of the obtained Raman spectra for (**A**) the range of the main band and (**B**) the Q-range. An example of the complete untreated Raman spectrum is shown in the inset. The red line displays the used baseline correction for each range indicated by the blue boxes. Effects of higher cooling rates (*q_g_* ↑) are indicated by the shown arrows: horizontal for shifts and vertical for intensity changes in the corresponding direction of the displayed arrow.

*σ_MB_* and *MP_Q_* demonstrate an identical shift to lower wavenumbers with increasing cooling rate. With increasing cooling rate, the intensity in the range from 510 to 610 cm^−1^ grows at the expense of the intensity of the 620 to 710 cm^−1^ range inducing the observed trend in *σ_MB_* (see also Figure A1A). A slight band increase centered at 540 cm^−1^ is observable while the band maximum shifts from 565 to 562 cm^−1^. The shift could point to an increase of *Q*^3^ units within the glass network with increasing cooling rate. The increase of the band centered at 540 cm^−1^ could relate on this trend and indicate a wider distribution of the bonding angle within the *Q*^3^ unit structures [33]. Further, a slight increase of intensity of the 600 cm^−1^ band is visible. If the 600 cm^−1^ band is assigned to *Q*^2^ units, a simultaneous increase with *Q*^3^ units would contradict recent reports in literature observing this range after permanent densification [34,35,40]. Therefore, the assignment of this band to Si-membered rings or defect bands sounds more suitable. In the range from 620 to 710 cm^−1^, an intensity decrease is observed within a band centered at 665 cm^−1^. A specific assignment of the 665 cm^−1^ band is, to our knowledge, not yet reported in the literature. Data using the *σ*-parameter of the main band subjected to different cooling rates has not been previously reported. Consequently, we cannot make a direct comparison between our observations and those reported in the literature.

Observing the changes of the Q-range, Tan et al. [41] previously reported a growth of the band at 950 cm^−1^ at the expense of the band at 1100 cm^−1^ with increasing cooling rate. Assigning *Q*^3^ species to the 1100 cm^−1^ band and *Q^2^* species to the 950 cm^−1^ band, they concluded a behavior following 2·*Q*^3^ ⇔ *Q*^2^ + *Q*^4^ with increasing cooling rate. In our work, we observe a smaller change of ratio for the 950 cm^−1^ band compared to the 1100 cm^−1^ band than [41] reported (see also Figure A1B). Our results are similar to the findings of [53] for the intensity ratio of *Q*^2^/*Q*^3^ indicating a small increase of the *Q*^2^ species with increasing cooling rate. Due to possible errors with a Gaussian deconvolution of the spectra and spectral noise dependent miscalculations, we want to focus in our work on the *maximum position* of the 1100 cm^−1^ band. We observe a linear shift of the *MP_Q_* to lower wavenumbers with increasing cooling rate. A shift of the *MP_Q_* to lower wavenumbers indicates an increase of the Si–O bond length within the *Q*^3^ species, related with a less dense network, based on an increasing cooling rate rather than a transition to *Q*^2^ species [39,54].

Using a linear fit to relate the behavior of *σ_MB_* and *MP_Q_* to the cooling rate, Equations (5) and (6) are determined.

(5) $$\log_{10}\left\{\frac{q_{g}\left(\sigma_{MB}\right)}{K/\min}\right\}=\frac{\sigma_{MB}-\left(587.0\pm 0.2\right)\left({\mathrm{cm}}^{-1}\right)}{\left(-0.42\pm 0.01\right)\left({\mathrm{cm}}^{-1}\right)}$$

(6) $$\log_{10}\left\{\frac{q_{g}\left(MP_{Q}\right)}{K/\min}\right\}=\frac{MP_{Q}-\left(1095.7\pm 0.2\right)\left({\mathrm{cm}}^{-1}\right)}{\left(-0.40\pm 0.04\right)\left({\mathrm{cm}}^{-1}\right)}$$

Since *q_g_*1 and *q_g_*2 were completely free of any residual stress, the depicted Equations (5) and (6) are only valid in a stress-free regime. A shift of −0.42 cm^−1^ and −0.40 cm^−1^ per order of magnitude of cooling rate was calculated for *σ_MB_* and *MP_Q_*, respectively. The linear trend of *MP_Q_* shown in Equation (6) is in good agreement with literature [53,55].

Figure 5B shows the behavior of *σ_MB_* and Figure 5D the behavior of *MP_Q_* versus uniaxial stress for US_T_g_1 and US_T_g_2 as well as US_annealed. The fit of *σ_MB_* and *MP_Q_* versus uniaxial stress for US_T_g_1 and US_T_g_2 are described using Equations (7) and (8).

(7) $$US\left(\sigma_{MB}\right)=\frac{\sigma_{MB}-\left(586.7\pm 0.2\right)\left({\mathrm{cm}}^{-1}\right)}{\left(-0.74\pm 0.17\right)\left(\frac{{\mathrm{cm}}^{-1}}{\mathrm{GPa}}\right)}$$

(8) $$US\left(MP_{Q}\right)=\frac{MP_{Q}-\left(1095.5\pm 0.2\right)\left({\mathrm{cm}}^{-1}\right)}{\left(-3.02\pm 0.26\right)\left(\frac{{\mathrm{cm}}^{-1}}{\mathrm{GPa}}\right)}$$

As in situ observations were performed during loading and unloading of the samples, we found that the same uniaxial stress conditions resulted in identical shifts for all observables. No hysteresis and, therefore, no permanent densifications are observed. This confirms that all observations up to −0.85 GPa were collected in the elastic regime of the glass. This is in good agreement with the findings of [40], reporting the elastic threshold for window glass above 6.5 GPa in hydrostatic conditions. Therefore, the loading and unloading cycles were simultaneously fitted to reduce the error. For *σ_MB_*, a change of −0.74 cm^−1^ per GPa is calculated. For *MP_Q_*, a change of −3.02 cm^−1^ per GPa is calculated.

**Figure A2:**
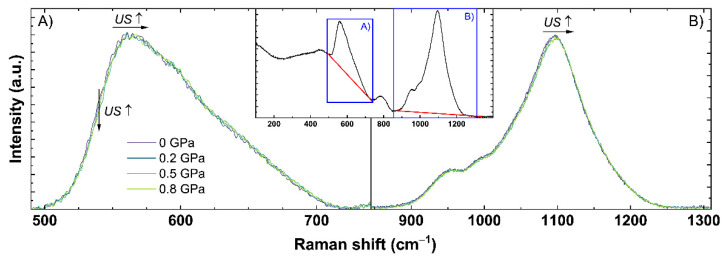
Detailed analysis of the obtained Raman spectra for (**A**) the range of the main band and (**B**) the Q-range. An example of the complete untreated Raman spectrum is shown in the inset. The red line displays the used baseline correction for each range indicated by the blue boxes. Effects of applied uniaxial stress (*US* ↑) are indicated by the shown arrows: horizontal for shifts and vertical for intensity changes in the corresponding direction of the displayed arrow.

Compared to *q_g_*1 and *q_g_*2, *σ_MB_* of US_T_g_1 and US_T_g_2 shows, within the elastic regime, only a minor shift to higher wavenumbers with increasing uniaxial stress. The small shift in our work could indicate just a change of the angle distribution of the Si–O–Si symmetric stretching vibrations (see also Figure A2A). It can be possible that the applied uniaxial stress in the elastic regime is not inducing enough energy into the network structure to force a transition of *Q*^3^ species to *Q*^2^. Comparing US_T_g_1 and US_T_g_2 (see Figure 5B) to US_annealed, the same linear behavior with a constant offset of 2 cm^−1^ is visible. This indicates that the network response to uniaxial stress in the elastic regime does not depend on the glass thermal history within our observed range.

*MP_Q_* changes significantly under the applied uniaxial stress. In Figure 5D, a linear shift of the *MP_Q_* to higher wavenumbers with increasing uniaxial stress can be observed. A detailed analysis of our Raman spectra shows no changes within the *Q*^1^, *Q*^2^ and *Q*^4^ bands, which are here strongly convoluted with the *Q^3^* band (see also Figure A2B). A shift of the *Q*^3^ band indicating a change of the mean Si-O bond length is clearly observed and well followed by the *MP_Q_*-parameter. Thus, this shift to higher wavenumbers is in good agreement with the expected compressive effects and happens due to a decrease of the interatomic distances under uniaxial stress. The samples with different thermal history, compared in Figure 5D, show a parallel behavior. The difference of behavior can be reduced to a constant offset given by Equation (6).

### 3.2. Calibration of the Brillouin Response

**Figure 6:**
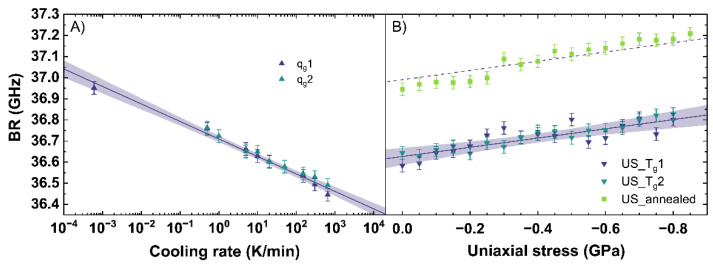
*Brillouin shift* (*BR*) in backscattering geometry with an excitation at 488 nm versus (**A**) cooling rate (*q_g_*) and (**B**) uniaxial stress (*US*). *q_g_*1 and *q_g_*2 are indicated by the dark blue and cyan up triangles. US_T_g_1 and US_T_g_2 are displayed by the dark blue and cyan down triangles. US_annealed is indicated by light green squares. The shaded areas show the 95% confidence interval of the linear fits.

Figure 6 shows the evaluation of the *BR* observable depending on different cooling rates (see Figure 6A) and under different uniaxial stress (see Figure 6B). Similar to the Raman observables, the *BR* shows linear trends.

With increasing cooling rate, the *BR* shifts to lower frequencies. The behavior of *BR* for *q_g_*1 and *q_g_*2 can be expressed with Equation (9).

(9) $$\log_{10}\left\{\frac{q_{g}\left(BR\right)}{K/\min}\right\}=\frac{BR-\left(36.71\pm 0.03\right)\left(\mathrm{GHz}\right)}{\left(-0.083\pm 0.003\right)\left(\mathrm{GHz}\right)}$$

According to the fit, a decrease of *BR* of −0.083 GHz per order of magnitude of cooling rate is measured. This trend is based on a rearrangement of the network due to a different cooling rate, modifying the Si-O-Si bond angle distribution and thus influencing the elastic properties of the network [56]. For soda–lime silicates, a higher cooling rate results in a less dense glass network resulting in a *BR* of lower frequency. Within the range of uncertainty, Raffaëlly [55] reported an identical behavior of *BR* with increasing cooling rate.

With increasing uniaxial stress, *BR* slightly shifts to higher frequencies in agreement with the increase of density. The fit of US_T_g_1 and US_T_g_2 under uniaxial stress is given in Equation (10).

(10) $$US\left(BR\right)=\frac{BR-\left(36.63\pm 0.03\right)\left(\mathrm{GHz}\right)}{\left(-0.22\pm 0.04\right)\left(\frac{\mathrm{GHz}}{\mathrm{GPa}}\right)}$$

For uniaxial compression, an increase of *BR* of −0.22 GHz per GPa is measured. Comparing our evaluations of *BR* to Tran [57], an identical slope, but a general offset of 2 GHz, can be observed. This offset is based on the different excitation wavelength used in both studies, and a correction from 514.5 nm to 488 nm removes the offset completely. The identical trend of our evaluations and that of Tran [57] for *BR* can be observed from 0 to 3.1 GPa, corresponding with [40] reporting the elastic regime up to 6.5 GPa. Similar to the stress observations of the Raman signal, an almost constant offset of 0.4 GHz between US_T_g_1 and US_annealed is visible. This confirms the assumption that the behavior of the network under uniaxial stress and within the elastic regime seems to be independent from the initial state of the glass network and therefore from the thermal history in our observed range.

### 3.3. Calibration of the Nd^3+^ Photoluminescence Response

**Figure 7:**
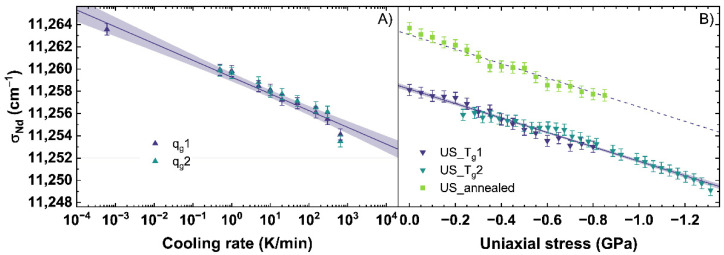
Parameter *σ_Nd_* corresponding to the ^4^F_3/2_→^4^I_9/2_ transition observed under an excitation at 780.42 nm (**A**) versus a change of cooling rate (*q_g_*) and (**B**) uniaxial stress (*US*). *q_g_*1 and *q_g_*2 are indicated by the dark blue and cyan up triangles. US_T_g_1 and US_T_g_2 are displayed by the dark blue and cyan down triangles. US_annealed is indicated by light green squares. The shaded areas show the 95% confidence interval of the linear fits.

The emission of Nd^3+^ was observed with an excitation at 780.42 ± 5 nm. Since this emission can depend on the excitation, the future users of this calibration must be careful if they work with a laser of another wavelength. The evolution of the parameter *σ_Nd_* corresponding to the ^4^F_3/2_→^4^I_9/2_ transition, is shown in Figure 7. With an increasing cooling rate, a shift of *σ_Nd_* from 11,264 cm^−1^ to 11,253 cm^−1^ is observed (see Figure 7A).

The change of *σ_Nd_* with different cooling rates is described with Equation (11), showing a decrease of the *σ_Nd_*-parameter of −1.51 cm^−1^ per order of magnitude of the cooling rate.

(11) $$\log_{10}\left\{\frac{q_{g}\left(\sigma_{Nd}\right)}{K/\min}\right\}=\frac{\sigma_{Nd}-\left(11259.3\pm 0.2\right)\left({\mathrm{cm}}^{-1}\right)}{\left(-1.51\pm 0.09\right)\left({\mathrm{cm}}^{-1}\right)}$$

Figure 7B depicts *σ_Nd_* under uniaxial stress, which decreases under compression from 11,264 cm^−1^ to 11,249 cm^−1^. The change of *σ_Nd_* under uniaxial stress can be described for the US_T_g_1 and US_T_g_2 with Equation (12).

(12) $$US\left(\sigma_{Nd}\right)=\frac{\sigma_{Nd}-\left(11258.2\pm 0.2\right)\left({\mathrm{cm}}^{-1}\right)}{\left(6.48\pm 0.14\right)\left(\frac{{\mathrm{cm}}^{-1}}{\mathrm{GPa}}\right)}$$

For this fit, a decrease of 6.48 cm^−1^ per GPa is calculated. The behavior of *σ_Nd_* is very different from the behavior of *σ_MB_*, *MP_Q_* and *BR*. The Raman and Brillouin observables were consistent with the effect of increasing density, i.e., increased for the glass with higher density not depending on the origin of the densification by lower cooling rate or higher uniaxial stress. However, *σ_Nd_* shifts to lower wavenumbers with an increase of both, cooling rate and uniaxial stress. This could indicate *σ_Nd_* depends on the state of disorder within the network structure. Both, a higher cooling rate as well as higher uniaxial stress, should increase the disorder state of the glass network [58,59]. Data using the *σ_Nd_*-parameter as a sensor for cooling rate or uniaxial stress in the elastic regime has not been previously reported. Therefore, we could not make a comparison of our data in the elastic regime and those reported in the literature. Nonetheless, comparisons to plastic deformed glass in [51,60] reported in the plastic regime a decreasing, albeit non-linear trend to lower wavenumbers with increasing permanent densification, which should also induce a higher disordered state in the glass network.

### 3.4. Non-Destructive Cooling Rate and Residual Stress Determination

Evaluating all calibrations obtained in this work, all observables (*σ_MB_*, *MP_Q_*, *BR* and *σ_Nd_*) show a slightly different behavior to a change of cooling rate or applied uniaxial stress. All observables shift with increasing cooling rates, but different slopes to lower wavenumbers. Applying uniaxial stress, *σ_MB_*, *MP_Q_* and *BR* shift with different slopes to higher wavenumbers in contrast to *σ_Nd_*, which shifts to lower wavenumbers. Based on these different behaviors, a comparison of the observables versus each other can quantify the independence of contributions of cooling rate or uniaxial stress on specific observables. On the one hand, the proposed comparison gives the possibility to distinguish the effects of the glass thermal history from those related to the applied uniaxial stress. On the other, it can be estimated which observation method or observable is suited to determine the cooling rate or the applied uniaxial stress.

**Figure 8:**
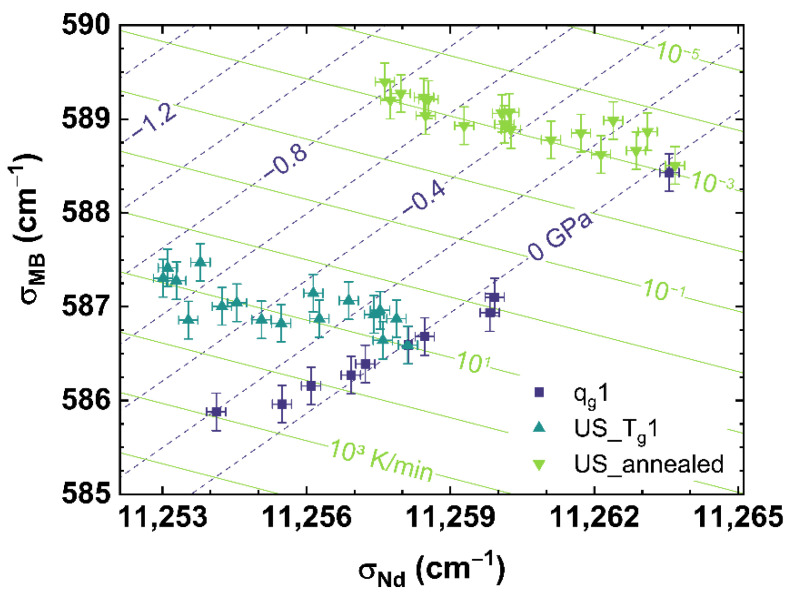
Observable comparison of *σ_MB_* over *σ_Nd_* for *q_g_*1 (dark blue squares), US_T_g_1 (cyan up triangles) and US_annealed (light green down triangles). The shown lines can be read as contour map based on the multiple linear regression models. The dark blue dashed lines indicate applied uniaxial stress, the light green lines indicate different cooling rates corresponding to the displayed values.

Figure 8 shows the comparison of *σ_MB_* to *σ_Nd_* for *q_g_*1 (see dark blue squares), US_T_g_1 (see cyan up triangles) and US_annealed (see light green down triangles). Figure 8 can be read as a contour map showing the corresponding cooling rates as light green contour lines versus the applied uniaxial stress shown as dark blue dashed contour lines. 

It can be observed, that US_T_g_1 and US_annealed intersect in their non-stressed state (0 GPa) with *q_g_*1 at the corresponding cooling rate, with which they were prepared. Corresponding to the behavior shown in Figure 5B, US_T_g_1 and US_annealed demonstrate an identical trend with increasing uniaxial stress.

The disparate slopes of *q_g_*1 and US_T_g_1 or US_annnealed, show a suitable independent contribution of cooling rate and uniaxial stress on *σ_MB_* or *σ_Nd_*, and therefore gives the possibility to separate the effects of cooling rate from that of applied uniaxial stress. The identical slopes of US_T_g_1 and US_annealed indicate a continuous behavior of the effect of uniaxial stress over the complete range of cooling rates observed in our work. Thus, we propose a multiple linear regression, interpolating the obtained experimental data to the full range of observation of this study, to calculate any combination of cooling rate and uniaxial stress by assuming orthogonality between the observables. The equations in the form

(13) $$Physical\;variable=a_{qg,\;\;US}\cdot Observable_{y}+b_{qg,\;\;US}\cdot Observable_{x}+c_{qg,\;\;US}$$

determining the cooling rate (*q_g_*) or the uniaxial stress (*US*) defined as *Physical variable* were prepared following [61] and

(14) $$\left[\begin{array}{cc} a_{qg} & a_{US}\\ b_{qg} & b_{US}\\ c_{qg} & c_{US} \end{array}\right]={\left(A^{T}\cdot A\right)}^{-1}\cdot A^{T}\cdot B$$

with $A=\left[\;\begin{array}{ccc} \underline{Observable_{y}} & \underline{Observable_{x}} & \underline{1} \end{array}\;\right]$ and $B=\left[\;\begin{array}{cc} \underline{\log_{10}\left(q_{g}\right)} & \underline{US} \end{array}\;\right]$. $\underline{Observable_{y}}$ and $\underline{Observable_{x}}$ represent column vectors of the chosen spectroscopic observables for comparison, i.e., *σ_MB_* versus *σ_Nd_*, with $\underline{\log_{10}\left(q_{g}\right)}$ and $\underline{US}$ as the related cooling rate and uniaxial stress column vectors (see Figure 8). $\underline{1}$ is a column vector of ones. All vectors are of the same length with the interrelated values ordered in the same rows. *A^T^* indicates the transposed matrix of *A* whereas *A*^−1^ is the inverse matrix of *A*. Equation (15) provides this multiple linear regression model to calculate the cooling rate and the uniaxial stress for the *σ_MB_* versus *σ_Nd_* comparison. All equations are only valid for the used glass composition and for a cooling rate from 6 × 10^−4^ to 650 K/min or applied uniaxial stress from 0 to −1.3 GPa. The uncertainty is estimated using the residuals by comparing the measured observations to the prediction of the models. All experimental values used to calculate the proposed linear regression models can be found in Table A1, Table A2, Table A3 and Table A4.

(15) $$\begin{array}{c} \log_{10}\left\{\frac{q_{g}\left(\sigma_{MB},\sigma_{Nd}\right)}{K/\min}\pm 0.12\right\}=-1.55\left(\frac{1}{{\mathrm{cm}}^{-1}}\right)\cdot \sigma_{MB}-0.21\left(\frac{1}{{\mathrm{cm}}^{-1}}\right)\cdot \sigma_{Nd}+3232.44\\ US\left(\sigma_{MB},\sigma_{Nd}\right)\pm 0.03\left(\mathrm{GPa}\right)=-0.28\left(\frac{\mathrm{GPa}}{{\mathrm{cm}}^{-1}}\right)\cdot \sigma_{MB}+0.10\left(\frac{\mathrm{GPa}}{{\mathrm{cm}}^{-1}}\right)\cdot \sigma_{Nd}-970.28\left(\mathrm{GPa}\right) \end{array}$$

**Figure 9:**
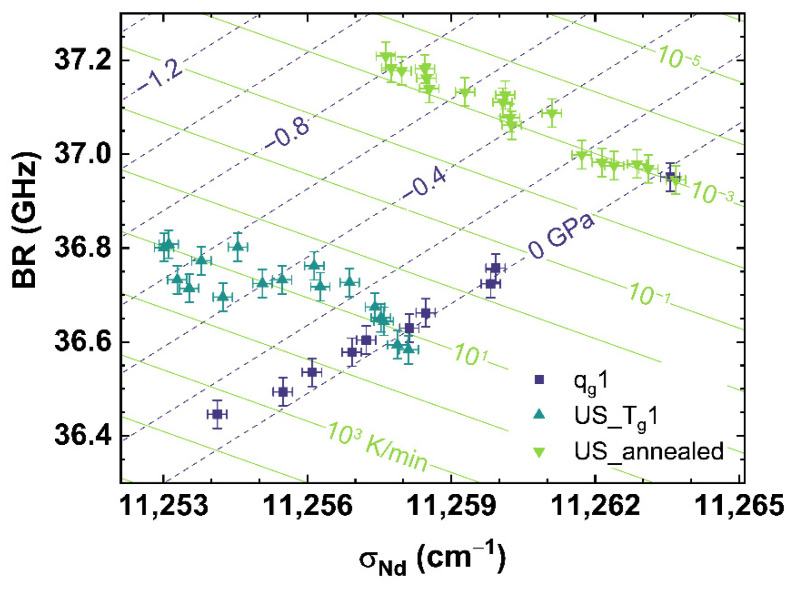
Observable comparison of *BR* over *σ_Nd_* for *q_g_*1 (dark blue squares), US_T_g_1 (cyan up triangles) and US_annealed (light green down triangles). The shown lines can be read as contour map based on the multiple linear regression models. The dark blue dashed lines indicate applied uniaxial stress, the light green lines indicate different cooling rates corresponding to the displayed values.

Figure 9 shows the comparison of *BR* to *σ_Nd_* for *q_g_*1 (see dark blue squares), US_T_g_1 (see cyan up triangles) and US_annealed (see light green down triangles). The *BR*-parameter shows a similar behavior as *σ_MB_*, although it is deduced from the Raman signal (see Figure 8). A detailed analysis of US_T_g_1 displays a S-shaped behavior with increasing uniaxial stress. This could be an artefact based on an inhomogeneous stress distribution inside the sample. The spectroscopic setup monitors only a relatively small volume depending on the focal spot of the laser, which was placed close to the surface of the sample. The increasing uniaxial stress could induce fluctuations of the stress field inside the sample cylinder due to shear stress, surface roughness, chippings or other inhomogeneities of the sample. A gradient of the cooling rate from the inside to the outside of the sample, also having a strong influence on the homogeneity of the stress distribution, was excluded due to the annealing process at *T_g_*. Comparing US_annealed to US_T_g_1, a pure linear behavior with increasing stress is visible for US_annealed, indicating that the S-shaped behavior of US_T_g_1 is an artefact of the measured sample or caused by higher cooling rates.

Equation (16) provides the multiple linear regression model to calculate the cooling rate and the uniaxial stress for the comparison of *BR* over *σ_Nd_*.

(16) log10{qg(BR,σNd)K/min±0.13}=−7.61(1GHz)·BR−0.28(1cm−1)·σNd+3398.57 US(BR,σNd)±0.03(GPa)=−1.38(GPaGHz)·BR+0.09(GPacm−1)·σNd−939.12(GPa)

**Figure 10:**
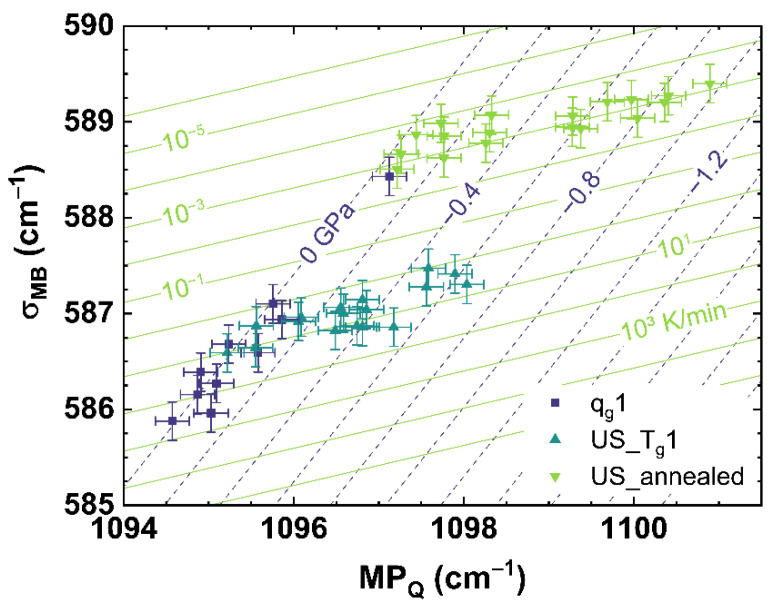
Observable comparison of *MP_Q_* over *σ_Nd_* for different cooling rates (dark blue squares, *q_g_*1) and under different uniaxial stress. The cyan up triangles show the cylinder cooled with 10 K/min (US_T_g_1), the light green down triangles indicate the cylinder cooled with 6 × 10^−4^ K/min (US_annealed). The dark blue dashed lines indicate a translation of the trend of cooling rate to different uniaxial stress shown by the corresponding numbers. The light green lines indicate a translation of the trend of uniaxial stress to different cooling rates shown by the corresponding numbers.

Figure 8 and Figure 9 show the comparison of observables deduced from different spectroscopic techniques, i.e., Raman spectroscopy versus photoluminescence and Brillouin spectroscopy versus photoluminescence, to separate the effects of cooling rate from those of applied uniaxial stress. However, we found it also possible to separate both effects by comparing suitable observables deduced from only one spectroscopic technique like Raman spectroscopy, which is shown in Figure 10 comparing *σ_MB_* to *MP_Q_*.

The approach comparing only observables deduced from the Raman spectrum shows a similar disparate behavior between *q_g_*1 and US_T_g_1 or US_annealed as *σ_MB_* versus *σ_Nd_* (see Figure 8) and *BR* versus *σ_Nd_* (see Figure 9). Using multiple linear regression, Equation (17) is calculated to estimate the cooling rate and the uniaxial stress for the comparison of *σ_MB_* over *MP_Q_*.

(17) log10{qg(σMB,σNd)K/min±0.18}=−2.58(1cm−1)·σMB+0.54(1cm−1)·MPQ+922.74 US(σMB,σNd)±0.05(GPa)=0.28(GPacm−1)·σMB−0.31(GPacm−1)·MPQ+174.50(GPa)

Figure 10 shows that our method of suitable observable comparison is also functional using just one spectroscopic observation technique, but at the expense of a higher uncertainty of this approach than the comparison of observable from two different spectroscopic techniques. However, this could be also based on the observables *σ_MB_* and *MP_Q_* itself. *σ_MB_* observes the behavior of the Raman spectrum in a range from 500 to 730 cm^−1^, resulting in a stable evolution of the parameter for a change of cooling rate or different uniaxial stress. This could be seen at the comparatively smaller error of the fit of *σ_MB_* compared to *MP_Q_* (see Equations (5)–(8)). However, observing a range of the Raman spectrum decreases the specificity to small changes compared to monitoring a single band contribution or *maximum position* like *MP_Q_*. The effects of cooling rate and uniaxial stress can have similar evolutions in the Raman spectrum (see Figure A1 and Figure A2), and therefore it is crucial to deduce suitable observables from the Raman spectrum that describe only the effects of cooling rate or applied uniaxial stress. Comparing only optical (Raman) versus acoustic (Brillouin) light scattering, similar to the approach comparing Raman and Brillouin scattering versus Nd^3+^ photoluminescence, we observed no distinguishable behavior related only to cooling rate or applied uniaxial stress. This suggests that the two types of measurements are here sensitive to the same underlying structural modifications induced by changing the cooling rate or the uniaxial stress. The distribution of the angle Si–O–Si, directly observed by Raman spectroscopy, could indeed also affect the elastic properties in the same proportion. Thus, for our provided methods to characterize the network structure, regarding the applied cooling rate and uniaxial stress, the inclusion of REE like Nd^3+^ as a sensor is recommended. Other rare-earth elements like Eu^3+^, Pr^3+^ or Sm^3+^, providing a photoluminescence, can also be utilized as sensors to monitor cooling rate or stress of the glass network structure [47,60,62].

The multiple linear regression models provided in our work relate a change of cooling rate to uniaxial stress. To perform a full 3D analysis of the glass network structure, regarding the cooling rate and residual stress, a correlation of uniaxial stress to isostatic stress is necessary. Furthermore, the effect of elasticity and plastic deformation has to be considered, especially in the transition range from elastic to plastic rearrangements of the glass network structure.

## 4. Conclusions

In our work, soda–lime silicate (OptiWhite) was observed cooled at different rates (6 × 10^−4^ to 650 K/min) and under applied uniaxial stress (0 to −1.3 GPa). Different observables were deduced from the spectra obtained by Raman, Brillouin and Nd^3+^ photoluminescence spectroscopies to identify changes in the glass network based on a change of cooling rate or uniaxial stress. Regarding the Raman signal, the barycenter shift of the main band and the *maximum position* of the Q-range were found to be suitable to monitor the response of the glass network. For the Brillouin spectroscopy, the center position of the Brillouin shift was utilized. Regarding the Nd^3+^ photoluminescence, the barycenter wavenumber of the ^4^F_3/2_→^4^I_9/2_ transition was used. All observables were related to the applied cooling rates or the applied uniaxial stress to provide interrelated calibration curves. These curves show a similar behavior compared to existing literature. The optical and acoustic observables show linear behaviors with both, increasing cooling rate and uniaxial stress, in good agreement with their expected variation of density. In contrary, the *σ_Nd_* presents a linear behavior to lower wavenumbers with increasing cooling rate and uniaxial stress following a structural disordering trend and not a density one. These differences in behavior allows us by comparison of different observables to establish a method able to distinguish a cooling rate effect from a uniaxial stress one. It is possible then to analyze the applied cooling rate and the uniaxial stress simultaneously and separate both influences. The provided calibrations were used to calculate multiple linear regression models of different observable comparisons. These established models can quantify the independence of contributions of cooling rate and uniaxial stress on specific observables and qualify the suited observables to determine the cooling rate and uniaxial stress. Further, they may be used as non-destructive characterization for soda–lime silicates with unknown thermal history and residual stress.

## Data Availability

The full data sets are contained within the article.

## Appendix A

**Table A1:** Experimental data and spectroscopic observables of the *q_g_*1 sample used for the multiple linear regression model.

log_10_(*q_g_*)	*US*	*σ_MB_*	*MP_Q_*	*BR*	*σ_Nd_*
(K/min)	(GPa)	(cm^−1^)	(cm^−1^)	(GHz)	(cm^−1^)
±0.01	±0.001	±0.2	±0.2	±0.03	±0.2
−3.222	0	588.4	1097.1	36.95	11,263.6
−0.301	0	587.1	1095.8	36.76	11,259.9
0.000	0	586.9	1095.9	36.72	11,259.8
0.699	0	586.7	1095.2	36.66	11,258.5
1.000	0	586.6	1095.6	36.63	11,258.1
1.301	0	586.4	1094.9	36.60	11,257.2
1.699	0	586.3	1095.1	36.58	11,256.9
2.176	0	586.2	1094.9	36.54	11,256.1
2.477	0	586.0	1095.0	36.49	11,255.5
2.813	0	585.9	1094.6	36.45	11,254.1

**Table A2:** Experimental data and spectroscopic observables of the *q_g_*2 sample used for the multiple linear regression model.

log_10_(*q_g_*)	*US*	*σ_MB_*	*MP_Q_*	*BR*	*σ_Nd_*
(K/min)	(GPa)	(cm^−1^)	(cm^−1^)	(GHz)	(cm^−1^)
±0.001	±0.001	±0.2	±0.2	±0.03	±0.2
−3.222	0	588.4	1097.1	36.95	11,263.6
−0.301	0	587.1	1095.8	36.76	11,259.9
0.000	0	586.9	1095.9	36.72	11,259.8
0.699	0	586.7	1095.2	36.66	11,258.5
1.000	0	586.6	1095.6	36.63	11,258.1
1.301	0	586.4	1094.9	36.60	11,257.2
1.699	0	586.3	1095.1	36.58	11,256.9
2.176	0	586.2	1094.9	36.54	11,256.1
2.477	0	586.0	1095.0	36.49	11,255.5
2.813	0	585.9	1094.6	36.45	11,254.1

**Table A3:** Experimental data and spectroscopic observables of the US_T_g_1 sample used for the multiple linear regression model.

log_10_(*q_g_*)	*US*	*σ_MB_*	*MP_Q_*	*BR*	*σ_Nd_*
(K/min)	(GPa)	(cm^−1^)	(cm^−1^)	(GHz)	(cm^−1^)
±0.001	±0.001	±0.2	±0.2	±0.03	±0.2
1	0.000	586.6	1095.2	36.58	11,258.1
1	0.050	586.9	1095.6	36.59	11,257.9
1	0.100	586.6	1095.6	36.64	11,257.6
1	0.150	587.0	1096.1	36.65	11,257.5
1	0.200	586.9	1096.1	36.67	11,257.4
1	0.250	587.1	1096.6	36.73	11,256.9
1	0.300	587.1	1096.8	36.76	11,256.1
1	0.350	586.9	1096.7	36.72	11,256.3
1	0.400	586.8	1096.5	36.73	11,255.5
1	0.450	586.9	1096.8	36.72	11,255.1
1	0.500	587.0	1096.9	36.80	11,254.6
1	0.550	587.0	1096.6	36.70	11,254.2
1	0.600	586.9	1097.2	36.71	11,253.5
1	0.650	587.5	1097.6	36.77	11,253.8
1	0.700	587.4	1097.9	36.81	11,253.1
1	0.750	587.3	1097.6	36.73	11,253.3
1	0.800	587.3	1098.0	36.80	11,253.0

**Table A4:** Experimental data and spectroscopic observables of the US_annealed sample used for the multiple linear regression model.

log_10_(*q_g_*)	*US*	*σ_MB_*	*MP_Q_*	*BR*	*σ_Nd_*
(K/min)	(GPa)	(cm^−1^)	(cm^−1^)	(GHz)	(cm^−1^)
±0.001	±0.001	±0.2	±0.2	±0.03	±0.2
−3.222	0.000	588.5	1097.2	36.95	11,263.7
−3.222	0.050	588.9	1097.4	36.97	11,263.1
−3.222	0.100	588.7	1097.3	36.98	11,262.9
−3.222	0.150	589.0	1097.7	36.98	11,262.4
−3.222	0.200	588.6	1097.8	36.98	11,262.1
−3.222	0.250	588.9	1097.8	37.00	11,261.7
−3.222	0.300	588.8	1098.3	37.09	11,261.1
−3.222	0.350	588.9	1098.3	37.06	11,260.3
−3.222	0.400	589.1	1098.3	37.08	11,260.2
−3.222	0.450	588.9	1099.3	37.13	11,260.1
−3.222	0.500	589.1	1099.3	37.11	11,260.1
−3.222	0.550	588.9	1099.4	37.13	11,259.3
−3.222	0.600	589.2	1099.7	37.14	11,258.5
−3.222	0.650	589.0	1100.0	37.16	11,258.5
−3.222	0.700	589.2	1100.0	37.18	11,258.4
−3.222	0.750	589.3	1100.4	37.18	11,258.0
−3.222	0.800	589.2	1100.4	37.18	11,257.7
